# Traffic as an Urban Health Determinant: Coverage of the New York City Congestion Pricing Plan on TikTok

**DOI:** 10.1007/s10900-024-01415-9

**Published:** 2024-10-25

**Authors:** Corey H. Basch, Helen Yousaf, Joseph Fera, Rafael Gonzalez Castillo

**Affiliations:** 1 Department of Public Health, William Paterson University, University Hall, Wayne, NJ 07470 USA; 2 Mailman School of Public Health, Columbia University NY, NY, 10032 USA; 3 Department of Mathematics, Lehman College, The City University of New York Bronx, NY, 10468 USA

**Keywords:** TikTok, Congestion Pricing, New York City, Urban Health

## Abstract

Social listening can provide deeper insight into public sentiment regarding policy proposals, as seen with the congestion pricing initiative in New York City (NYC). The purpose of this study was to assess the content of a sample of TikTok videos related to congestion pricing in NYC. A total of 100 unique videos were extracted from the hashtag #nyccongestionpricing on TikTok and coded for the presence of the following information based on four different areas of focus: video metrics, details about pricing plan, effects of pricing plan, and user-related issues. In total, the 100-video sample received 17,375,818 views, 1,285,610 likes, 89,883 favorites, and 100,634 comments. Only 3 of the 25 content characteristics were present in a majority (> 50) of the videos. These were mentions location (below 60th street of Manhattan) (*N* = 65), mentions cost (*N* = 61), and mentions cons of pricing (*N* = 56). Mentioning location had a significant effect on the views (*p* = 0.0163) and likes (*p* = 0.0225) a video received. Mentioning cost had a statistical impact on the views (*p* = 0.0098), likes (*p* = 0.0128), favorites (*p* = 0.0128), and comments (*p* = 0.0030) that the video received. Mentioning the MTA and benefits of the congestion plan significantly lowered metrics of engagement. Themes that emerged were personal, often negative and tended to focus more on cost to citizens and location, despite the evidence of negative public health impacts. This research can be used as a springboard for future research that examines social listening at the intersection of public health and policy.

## Introduction

Traffic congestion and transit-related issues are key determinants of urban health [1], primarily due to their by-products such as poor air quality, pedestrian safety concerns [2], vehicular accidents [3–4], and noise pollution [5–6], which impact health in both the short- and long-term [1, 7]. Additionally, traffic congestion can alter the built environment [8], exacerbate equity issues [9], and serve as a critical economic concern [10]. Traffic congestion is a significant contributor to global climate change [11], and more specifically to exhaust and non-exhaust air pollution [12].

Those dwelling in urban environments like New York City (NYC), the most populous and among the most-dense cities in the United States, face unique transit related challenges [13]. With a population over 8.5 million people, the density of the city is marked by an increase of nearly 800,000 in its population since the 1950s [14], and is further influenced by a swarm of commuters who reside in nearby New Jersey, Connecticut, and Long Island [15]. As such, the health of both NYC residents and commuters is influenced by a variety of factors including: vehicle-pedestrian interactions among its denizens, walkability, a varied set of stressors, and exposure to environmental health pollutants [16].

NYC has a history of issues related to air pollution. Following the 1966 NYC Smog, which caused dangerously high levels of sulfur dioxide in the air, it was labeled the most polluted city in the United States in 1967 [17]. In response, a series of national and state legislative actions, along with social movements, emerged with the aim of addressing the impacts of past environmental disasters and preventing future occurrences [17]. Regardless of these efforts, nearly 2,400 NYC residents die annually as a result of ozone and particulate matter (PM2.5) exposure [18]. Further, NYC health impact reports estimate that nearly 2,000 deaths and 1,400 admissions into the hospital for lung and heart conditions are caused by fine particle pollution annually [19]. The most prevalent gasses in NYC air have been identified as nitrogen, sulfur, carbon oxides, ozone, methane, and other hydrocarbons [20], which are known to cause cancer, cardiovascular disease, and impacts to the reproductive and nervous systems [21–22].

Policy-based interventions have been used to improve traffic-related air quality, but research shows these strategies are diverse and often encounter barriers. Khreis et al. found that interdisciplinary approaches, such as behavioral changes and land use planning, are often overlooked in favor of traditional, supply side solutions by transportation authorities [23]. In the United States, traffic congestion policies vary widely due to differing regional and political factors. Alongside air quality initiatives, NYC has focused on road safety with the implementation of Vision Zero in 2014, aimed at reducing traffic-related incidents and fatalities. The program saw reductions to the city speed limit, implementation of speed cameras, and health education campaigns citywide [24–25]. Despite this, from January 1 to June 30, 2024, there have been 18,223 motorist, 2,062 cyclist, and 4,323 pedestrian injuries across the city, with 51 motorist, 12 cyclist and 61 pedestrian fatalties [26].

Congestion pricing is an increasingly popular public policy proposal worldwide, with the goal of mitigating the consequences of traffic in high-congested urban areas [27]. Proponents of congestion pricing laws cite its previous success in addressing traffic-related health in other metropolitan cities around the world, like London, Singapore, and Stockholm [28]. Others raise concerns that such policies disproportionately impact low-income commuters who cannot afford to pay extra to drive to work or school [29–30]. Within a community health context, congestion pricing plays an important role because increased vehicular traffic is associated deleterious health outcomes such as asthma, cardiovascular disease, lung disease, and cancer [31].

The latest congestion pricing plan, introduced under New York Governor Kathy Hochul, mirrors the proposal made by then-Mayor Michael Bloomberg in 2007 as part of his PlaNYC [32]. Recently, the plan received approval from the Federal Highway Administration under President Joe Biden and was set to be implemented in June 2024 [33]. The goals of the program were three-fold: (1) relieve congestion and traffic, (2) decrease greenhouse gas emissions, and (3) improve safety of the streets [34]. MTA officials also noted that consumers would benefit from nearly 100,000 fewer vehicles a day in the impacted zone and that money from the tax would go towards strengthening public transportation in the city [35]. Amidst support from federal authorities, the plan was met with backlash from local political leaders, who filed lawsuits against the plan, citing strains on the environment and traffic of nearby regions [36]. What followed was a sudden reversal of plan by the New York Governor just two weeks before the date of effect [24].

Previous research has exclusively focused on the economic, political, and health implications of congestion pricing plans [37]. The NYC congestion pricing plan could significantly impact the daily lives and health of constituents, highlighting the importance for public and health officials to understand the full range of public opinion. To our knowledge, these sentiments have not been systematically captured, regardless of being widely shared in different arenas such as social media.

TikTok, with one billion users worldwide [38], maintains a prominent hold as an opinion-sharing platform [39], and is known for its powerful algorithm which maximizes data on prior engagement to suggest new content [40–43]. For issues like the congestion pricing plan which concerns the quotidian life of everyday citizens, understanding public opinions shared on social media can be a powerful indicator of whether public officials will see their policy through. The purpose of this study was to assess the content of a sample of TikTok videos related to congestion pricing in NYC.

## Methods

The methods for this study were based on prior research [44]. A total of 100 unique videos were extracted from the hashtag #nyccongestionpricing on TikTok and watched in their entirety. This hashtag was selected as it captured the most viewed videos related to the New York City congestion pricing plan at the time of data collection in June 2024. Each video was watched and coded for the presence of the following information based on four different areas of focus:

1. Video metrics: This included the number of source followers; perceived gender of person in video; view, like, comment, and bookmark count; length of video; use of humor; and additional hashtags tagged.
2. Details about pricing plan: Videos were examined for content about the cost; locations impacted; exemptions to pricing plan; times in effect; date of effect; method drivers will be charged; map of impacted zone; intended goals of plan; research mentioned; and inclusion of news clippings.
3. Effects of pricing plan: Content was coded for mentions of overall benefits versus cons; environmental benefits; health impacts of traffic congestion; administrative responses; anecdotal complaints; impact on walkers, bikers, public transportation; impact on quality of life; effect on urban landscape.
4. User-related issues: Encompassing this category were MTA issues; impact on commuters versus residents; professional groups impacted; recommendations from transportation, government, or health officials; suggestions for alternate modes of transportation; additional resources provided.

Videos were coded both qualitatively for descriptions related to each content category and deductively (0 = absent and 1 = present). One hundred TikTok videos on congestion pricing were analyzed in this study. One person (HY) reviewed all 100 videos and identified the presence of predetermined content. A second person (CB) reviewed a random sample of the videos for the same content. Out of 288 data points they collected, the reviewers disagreed on only 5 resulting in an interrater reliability score of κ = 0.9603. Descriptive statistics and independent t-tests (α = 0.05) were carried out with MS Excel. This study was determined to be non-human subjects by the Institutional Review Board at William Paterson University (# 2024 − 350).

## Results

In total, the 100-video sample received 17,375,818 views, 1,285,610 likes, 89,883 favorites, and 100,634 comments with respective averages (standard deviations) of: 173,758.18 (638,748.35), 12,856.10 (51,720.33), 898.83 (3,631.85), and 1,006.34 (3,107.17). The videos ranged in length from 10 s to 304 s with an average (standard deviation) length of 79.85 (49.87) seconds. Of the 100-video sample, 47% were against congestion pricing, 15% were in favor of it, and 38% were neutral on the issue.

Table 1 presents a listing of the prespecified content the reviewers looked for in the videos. The number of videos containing this content is given (N) along with the views, likes, favorites, and comments that videos with this content garnered. Relevant percentages are also included. Only 3 of the 25 content characteristics were present in a majority (> 50) of the videos. These were mentions of location (below 60th street of Manhattan) (*N* = 65), mentions cost (*N* = 61), and mentions cons of pricing (*N* = 56). Close to 50% of the videos sampled mentioned the MTA (*N* = 49) and mentioned the benefits of the pricing plan (*N* = 47). Less than 5% of the sample included additional resources for more information (*N* = 3) and mentioned the impact on bikers (*N* = 2). Only 22 videos mentioned environmental benefits. These videos received a total of 3,199,203 views (18.41% of all views), 127,016 likes (9.88% of all likes), 11,633 favorites (12.94% of all favorites), and 27,897 comments (27.72% of all comments).

**Table 1:** Observed content, views, likes, comments, and shares of 100 TikTok Videos on congestion pricing

	N	Views	%	Likes	%	Favorites	%	Comments	%
	100	17,375,818	100%	1,285,610	100%	89,883	100%	100,634	100%
Mentions location (below 60th street or Manhatten)	65	16,229,343	93.40%	1,209,722	94.10%	79,824	88.81%	86,103	85.56%
Mentions cost	61	16,520,344	95.08%	1,242,255	96.63%	86,991	96.78%	95,401	94.80%
Mentions cons of pricing plan	56	12,614,232	72.60%	801,283	62.33%	71,846	79.93%	67,804	67.38%
Mentions the MTA	49	2,428,676	13.98%	384,658	29.92%	8,600	9.57%	13,470	13.39%
Mentions benefits of pricing plan	47	3,732,693	21.48%	152,288	11.85%	13,170	14.65%	30,917	30.72%
Shows news clippings/news articles	37	10,946,648	63.00%	556,603	43.29%	46,936	52.22%	67,519	67.09%
Mentions legislative policies/administrative response	36	2,420,086	13.93%	386,831	30.09%	9,833	10.94%	18,989	18.87%
Explains intended goal of program	34	2,400,147	13.81%	100,083	7.78%	8,427	9.38%	15,144	15.05%
Mentions exemptions to rule	30	5,052,444	29.08%	499,717	38.87%	26,778	29.79%	48,773	48.47%
Mentions impact on public transportation	29	1,340,352	7.71%	58,350	4.54%	3,578	3.98%	8,761	8.71%
Mentions times (e.g. 5 am to 9 pm and 9–9 on weekends) in effect	28	12,272,217	70.63%	813,984	63.32%	71,995	80.10%	61,124	60.74%
Mentions date of effect	27	2,110,603	12.15%	358,580	27.89%	6,808	7.57%	12,578	12.50%
Mentions environmental benefits	22	3,199,203	18.41%	127,016	9.88%	11,633	12.94%	27,897	27.72%
Includes opinions/recommendations from transportation, government, or health officials	22	3,473,047	19.99%	128,735	10.01%	13,376	14.88%	27,634	27.46%
Shows map of zone impacted	19	5,749,897	33.09%	326,511	25.40%	28,271	31.45%	23,118	22.97%
Mentions MTA issues	17	1,043,499	6.01%	35,867	2.79%	2,649	2.95%	8,391	8.34%
Suggests alternate routes/mode of transportation	14	1,249,086	7.19%	67,410	5.24%	5,172	5.75%	7,282	7.24%
Impact on quality of life	13	9,193,013	52.91%	475,336	36.97%	39,151	43.56%	51,452	51.13%
Interviews other people for their opinion	12	3,485,595	20.06%	149,871	11.66%	17,329	19.28%	38,615	38.37%
Mentions how drivers will be charged (toll reader)	11	910,725	5.24%	316,145	24.59%	5,150	5.73%	9,472	9.41%
Mentions impacts of traffic, congestion (i.e. stress)	9	88,084	0.51%	3,610	0.28%	251	0.28%	586	0.58%
Uses Humor	7	3,054,085	17.58%	294,808	22.93%	22,898	25.48%	7,312	7.27%
Mentions improved urban landscape	5	789,304	4.54%	40,463	3.15%	4,288	4.77%	4,201	4.17%
Additional resources provided for more info	3	73,671	0.42%	3,338	0.26%	277	0.31%	490	0.49%
Mentions impact on bikers	2	41,654	0.24%	1,743	0.14%	138	0.15%	316	0.31%

For content present in nearly 50% or more of the sample, independent one-tailed t-tests (α = 0.05) were performed to determine if the presence of this content had a statistical effect on the views, likes, favorites, and comments they received. Table 2 presents the p-values obtained from these 20 tests; those with a p-value less than 0.05 were considered significant and are indicated with an *.

**Table 2:** Results from independent one-tailed t-tests (α = 0.05). An * indicates a statistically significant result

	p-value			
	Views	Likes	Favorites	Comments
Mentions location (below 60th street or Manhattan)	0.0163*	0.0225*	0.0601	0.0538
Mentions cost	0.0098*	0.0128*	0.0128*	0.0030*
Mentions cons of pricing plan	0.1622	0.3871	0.0945	0.2148
Mentions the MTA	0.0277*	0.1725	0.0249*	0.0102*
Mentions benefits of pricing plan	0.0741	0.0337*	0.0471*	0.1426

Mentioning location had a significant effect on the views (*p* = 0.0163) and likes (*p* = 0.0225) a video received. In both cases, the presence of this content resulted in a higher average of views (249,682.20 vs. 32,756.43) and a higher average of likes (18,611.11 vs. 2,168.23). Mentioning cost had a statistical impact on the views (*p* = 0.0098), likes (*p* = 0.0128), favorites (*p* = 0.0128), and comments (*p* = 0.0030) the video received. In all cases, the average was higher for videos featuring this content: views (270,825.31 vs. 21,935.23), likes (20,364.84 vs. 1,111.67), favorites (1,426.08 vs. 74.15), and comments (1,536 vs. 134.18). Mentioning the MTA had a significant effect on the views (*p* = 0.0277), favorites (*p* = 0.0249), and comments (*p* = 0.0102) that a video received. In these cases, videos mentioning the MTA received a lower average number of views (49,564.82 vs. 293,081.22), favorites (175.51 vs. 1,593.81), and comments (274.90 vs. 1,709.10). Mentioning the benefits of the pricing plan affected the number of likes (*p* = 0.0337) and favorites (*p* = 0.0471) a video received. In both cases, videos with this content had a lower average number of likes (3,240.17 vs. 21,383.43) and favorites (280.21 vs. 1,447,42).

## Discussion

The analysis revealed patterns in public discourse and levels of engagement on social media related to congestion pricing in NYC. The 100 videos included in this sample accumulated over 17 million views, 1.2 million likes, nearly 90,000 favorites, and more than 100,000 comments. Social listening, while often referred to in marketing contexts, has become increasingly operationalized in public health as a powerful tool in analyzing the prevalence and reach of social media content related to topics. The World Health Organization and the United States Centers for Disease and Control and Prevention have encouraged social listening as a methodology to gauge public engagement with health education information [45–46]. Keeping this in mind and given the acknowledgement of TikTok as a powerful platform in marketing campaigns [47], this work can serve as the foundation for providing well-rounded information related to transportation and urban health.

Videos that discussed the cost implications of congestion pricing had a statistically significant impact on the views. These averaged 270,825 views (16,520,344 cumulative total for the 61 videos), 20,364 likes, 1,426 favorites, and 1,536 comments, exceeding those that did not include this content. This indicates that economic factors associated with congestion pricing are likely propelling the discussion, as many residents view the added toll as an extra burden on top of the already high cost of living in NYC. The extent to which NYC residents perceive the benefits of the pricing plan to outweigh its drawbacks will likely depend on their socioeconomic status. In addition to being one of the most expensive cities to live in worldwide, NYC, and more specifically Manhattan, has one of the widest income gaps nationally [48]. The Gini coefficient (a measure of income inequality) for New York County was 0.60, placing it closer to the 1.0 end of the spectrum (maximum) as opposed to the minimum (0) [49].

Mentions of environmental benefits only appeared in 22 of the videos but had 3,199,203 views. Further, while they attracted a notable proportion of comments relative to other engagement metrics, they had a lower percentage of likes (9.88%) and favorites (12.94%). The results of the analysis indicate a relative lack of conversation around improving air quality, which is somewhat surprising. According to the greeNYC Behavioral Impact Study, New Yorkers self-identified as impactful actors in environmental change and roughly half of respondents (43%) reported high concern and low skepticism about the environment [50]. Moreover, it is reasonable to expect greater concern from New Yorkers about the environmental impact of new policies, especially in light of a recent report from the NYC Mayor’s Office of Climate and Environmental Justice, which identified Upper Manhattan as one of the neighborhoods most affected by environmental injustice and inequality [51].

Posts with references to the MTA received lower engagement, with average views dropping to 49,564 compared to 293,081 for videos that did not mention the MTA. Similarly, favorites (9.57%) and comments (13.39%) were substantially lower than for other categories. With nearly 472 stations, the subway averaged daily ridership of 3.2 million in 2022 [52]. Despite this dependence, many still fear the subway for safety concerns. According to a 2023 poll, only 49% and 22% of New Yorkers feel safe riding the subway during the day and at night, respectively [53]. The lack of sense of safety can be mainly attributed to the rise in violent incidents and other disturbances.

Content related to the specific location being below 60th Street in Manhattan showed a statistically significant increase in both views and likes, with averages of 249,682 views and 18,611 likes compared to videos that did not mention this location (32,756 views and 2,168 likes). According to NYC Planning, nearly 664,000 commuters travel to Manhattan for employment [54] which contributes to the nearly 1 million people who commute into the borough daily [55]. In addition, Manhattan below 60th Street is also famed for being the location of Broadway and Times Square, which are major sights of attraction and tourism.

This study is limited by its cross-sectional design which only captures the relevant TikTok content up to the point of data collection, and limits the generalizability of the findings. Future research can expand on this research by increasing the sample size, number of hashtags used in the search, and analyzing comments for additional insight. Despite these shortcomings, this study offers insight into sentiments aired on TikTok around congestion pricing in NYC.

Congestion pricing plans would be expected to have varying effects on people due to socio-economic and other factors [1]. Given this, policies like congestion pricing elicit both pros and cons, many of which are dependent on those impacted. The congestion pricing plan in NYC was intended to go into effect on June 30, 2024, however, the anticipated roll-out of the plan was reversed. This study revealed the aspects of congestion pricing that resonate most with viewers. Themes that emerged were personal, often negative and tended to focus more on cost to citizens and location, despite the evidence of negative public health impacts [56]. Given the unsuccessful implementation of the pricing plan, it warrants examining the degree to which negative public sentiment contributed to this reversal. In fact, in her announcement to indefinitely pause on congestion pricing, Governor Hochul acknowledged the potential economic burden on working-class families, an opinion shared by the majority of views in this sample [57]. These findings can be applied to future communications about congestion pricing with the aim of making them more well-rounded and relevant to a diverse population.

## Data Availability

Not applicable.

## Code Availability

Not available.
